# Central retinal vein occlusion in a young healthy COVID-19 patient: A case report

**DOI:** 10.1016/j.ajoc.2020.100992

**Published:** 2020-11-16

**Authors:** Tal Yahalomi, Joseph Pikkel, Roee Arnon, Yuval Pessach

**Affiliations:** Department of Ophthalmology, Samson Assuta Ashdod Hospital, Faculty of Health Sciences, Ben-Gurion University of the Negev, Israel

**Keywords:** COVID-19, CRVO, Central retinal vein oclusion

## Abstract

**Purpose:**

We detail a unique case of a healthy 33-year-old suspected COVID-19 patient who presented with unilateral Central Retinal Vein Occlusion, possibly as a complication of COVID-19.

**Observations:**

A 33-year-old healthy male was referred to the emergency department due to blurred vision in his left eye for the past month, accompanied by flashes of light without any accompanying neurological symptoms. The patient reported a three-week period of fatigue, dry cough, and shortness of breath ended about 2 weeks prior to the ocular symptoms. He was not tested for COVID-19 at the time of his respiratory complaints.

The clinical examination and the ancillary tests confirmed the diagnosis of a left eye Central Retinal Vein Occlusion.

During admission, a real-time reverse transcription-polymerase chain reaction (RT-PCR) for SARS-CoV-2 from a nasopharyngeal swab was performed and was found to be negative, however, an IgG/IgM Rapid Test (Inzek International Trading, the Netherlands) was performed and was found to be IgM negative and IgG Positive for SARS-CoV-2, confirming recovery from COVID-19.

**Conclusions and importance:**

To the best of our knowledge this is the first report of CRVO in association with COVID-19.

As the literature on human ocular manifestations of COVID-19 is still sparse, our case emphasizes the need for further investigation of ocular complication associated with this novel disease.

## 1 Introduction

Coronavirus disease (COVID-19), has been declared by the World Health Organization (WHO) as a pandemic on March 11th^,^ 2020.^1^

As of september 13th 2020, more than 28 million cases have been reported globally resulting in more than 900,000 deaths.^2^

The most common symptoms of COVID-19 include fever, dyspnea, dry cough, and loss of smell and taste. The progression of the disease could lead up to acute respiratory distress syndrome (ARDS), septic shock, and multi-organ failure.^3^

The pathogenesis of COVID-19 remains poorly understood, although there is increasing evidence that Inflammatory cytokine storm and viral evasion of cellular immune responses play a fundamental role in disease severity and progression.^4^Coagulopathy and disseminated intravascular coagulation (DIC) have been considered as important complications of severe COVID-19 and may be regarded the most common causes of death.^5^, ^6^, ^7^ COVID-19 may predispose patients to thromboembolic events, both in the arterial and venous circulations, due to excessive inflammation, endothelial dysfunction, platelet activation, and stasis.^6^

From the ocular perspective, up to this point, there have been reports of ocular infections presenting with conjunctivitis,^8^ or retinal microvascular changes such as retinal microangiopathy,^9^ cotton wool spots and microhaemorrhages,^10^ acute middle maculopathy and an acute macular neuroretinopathy,^11^ a Papillophlebitis,^12^ all presented in COVID-19 patients.

Central retinal vein occlusion (CRVO) is a prevalent retinal vascular disease that predominantly occurs in older individuals and results in a unilateral vision loss.

The pathogenesis of CRVO is believed to follow the principles of Virchow's triad for thrombogenesis, including vessel damage, stasis, and hypercoagulability. Age is the most important factor since the vast majority of cases occur in patients over the age of 60.

The occurrence of CRVO among persons under 50 years of age is considered to be rare and has been reported to be less than 1.5 cases per year at a single institution, or 0.93 per 1000 among persons under 64 years of age,^13^ while non ischemic CRVO among the younger population is accountable to be 60% of cases.^14^

We present a unique case of a healthy 33-year-old suspected COVID-19 patient who presented with unilateral CRVO, possibly as a complication of COVID-19.

## 2 Case report

A 33-year-old healthy male was referred to the emergency department due to blurred vision in his left eye for the past month, accompanied by flashes of light without any accompanying neurological symptoms.

Further investigation ruled out any medical details such as hypertension, obstructive sleep apnea, obesity or known hypercoagulability state. The patient reported a three-week period of fatigue, dry cough, and shortness of breath ended about 2 weeks prior to the ocular symptoms. He was not tested for COVID-19 at the time of his respiratory complaints. Familial and social history were noncontributory. The patient denied smoking or past use of drugs.

At presentation uncorrected visual acuity was 20/20 OD and 20/25 OS. Pupils were equal, round, and reactive to light with an afferent defect OS. Motility and confrontational visual fields were normal OU. He was able to identify 24/24 Ishihara color plates.

**Fig. 1:**
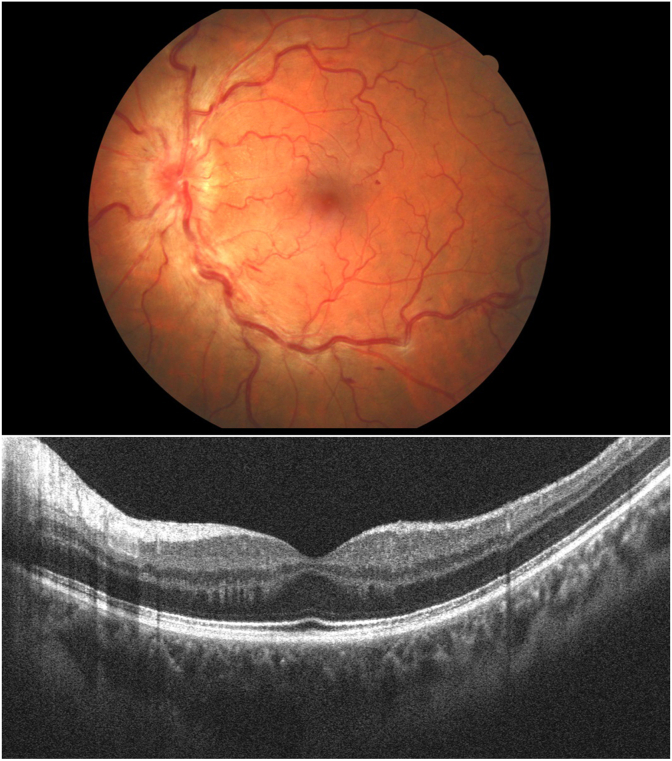
A- Fundus photography of the left eye showing tortuosity and dilatation of all branches of the central retinal vein, dot, blot and flame-shaped hemorrhages throughout all four quadrants, and optic disc edema. B- Optical Coherence Tomography (OCT) of the macula was within normal limits.

On Slit-lamp examination, the anterior segment was within normal limits, including intraocular pressure (IOP). Dilated fundus exam was within normal limits in the right eye. As for the left eye, fundoscopy showed tortuosity and dilatation of all branches of the central retinal vein, dot, blot and flame-shaped hemorrhages throughout all four quadrants, and optic disc edema (Fig. 1A).

Laboratory evaluation was unremarkable with no evidence of hypercoagulability markers such as aPTT (Activated partial thromboplastin time), PT (Pothrombin time), INR (International normalized ratio), LAC RVVT (Lupus anticoagulant Russell's Viper Venom Time), LAC SCT screen (Lupus anticoagulant Silica Clotting Time), Anti-Thrombin-III, LAC SCT ratio (Lupus anticoagulant Silica Clotting Time ratio), Protein C activity, Protein-S antigen, Anti Cardiolipin IgM and IgG, B2 Glycoprotein IgM and IgG, Fibrinogen and D-dimer were performed and were only slightly abnormal.

Neurological examination did not reveal focal neurological deficit. Computed tomography (CT) of the brain and orbits, CT angiography and CT venography showed no pathological findings.

Optical Coherence Tomography (OCT) of the macula was within normal limits (Fig. 1B), and Goldmann Perimetry showed normal visual fields OU.

**Fig. 2:**
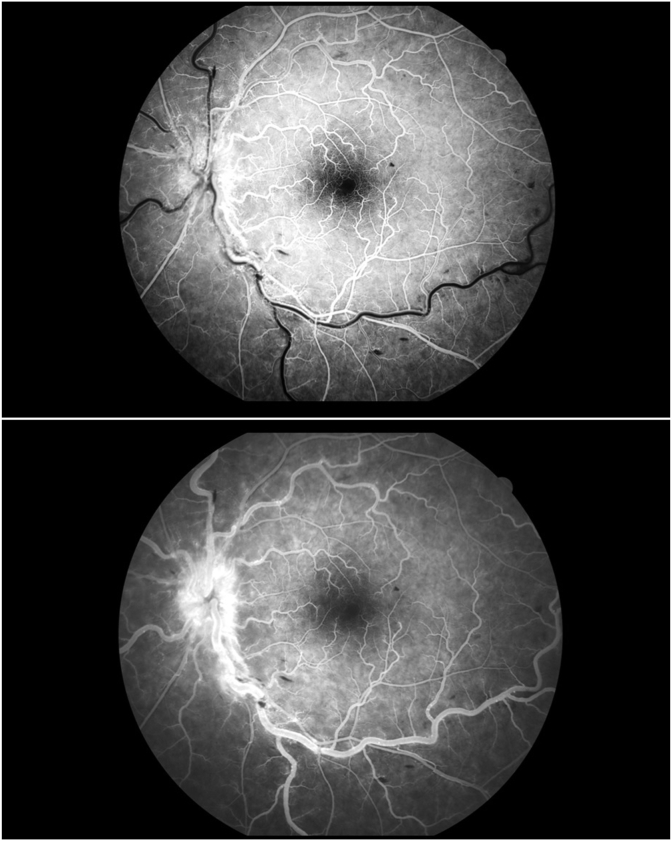
A- Fluorescein angiography of the left eye in 19 seconds showing marked delay in arteriovenous transit time, masking by retinal hemorrhages. B- Fluorescein angiography of the left eye in 47 seconds showing marked delay in venous filling, staining of dilated tortuous veins and masking by retinal hemorrhages, optic disc leakage. There isn't evidence of widespread capillary no perfusion areas. All well fit with non ischemic CRVO.

Fluorescein angiography showed marked delay in arteriovenous transit time, staining of dilated tortuous veins and masking by retinal hemorrhages. All well fit with non ischemic CRVO (Fig. 2A and B).

During admission, a real-time reverse transcription-polymerase chain reaction (RT-PCR) for SARS-CoV-2 from a nasopharyngeal swab was performed and was found to be negative, however, an IgG/IgM Rapid Test (Inzek International Trading, the Netherlands) was performed and was found to be IgM negative and IgG Positive for SARS-CoV-2, confirming recovery from COVID-19.

The patient was reevaluated at monthly follow-ups up to complete resolution of symptoms and gradually improvement of the retinal vascular appearance.

## 3 Discussion

To the best of our knowledge this is the first report of CRVO in association with COVID-19. As the diagnosis was done several weeks after the resolution of systemic and respiratory symptoms of COVID-19, we could not find SARS-CoV-2 RNA in his respiratory samples. Nevertheless, presence of a positive SARS-CoV-2-IgG COVID-19 Rapid Test (Inzek International Trading, the Netherlands) can serve as a suspicious basis for the diagnosis. The reported specificity of the kit that was used is 99.3% (IgG), and sensitivity of 97.4% (IgG),^15^ making a false-positive test result less likely. In addition, the onset of the patient's symptoms corresponded to the beginning of sustained and wide-spread community transmission of SARS-CoV-2 in Israel.^16^

The occurrence of CRVO in adults under 60 years of age has been reported to be considerably less common than in older patients. Systemic hypertension was found to be the most prevalent risk factor for CRVO in young patients, followed by diabetes mellitus and hyperlipidemia,^17^ all of the above were not found in our patient.

Nevertheless, hypercoagulability is often reported as a significant risk factor for CRVO in younger individuals, in particular, deficiencies of anticoagulant protein C, S, and antithrombin III and activated protein C resistance.

Work up for CRVO should include assessments of visual acuity, Fluorescein angiography, visual fields and if possible, electroretinography.

At follow-up, it is crucial to perform gonioscopy to check for angle neovascularization, and for patients under 50 years of age it is important to rule out thrombophilia.^18^

The mechanism responsible for COVID-19 thromboembolic events is still poorly understood, but may be due to excessive inflammation, hypoxia, immobilization, and DIC. Klok FA et al. found a 31% incidence of thrombotic complications in ICU patients with COVID-19 infections.^7^

Two major possible mechanisms has been offered to explain vascular damage in COVID-19 disease, first a pseudo-vasculitis state as a result of a viral infiltration of the endothelial cells,^19^ and second, a hypercoagulable condition, characterized by a disseminated intravascular coagulation-like (DIC-like).^20^

Those mechanisms could explain the association between the potential impact on retinal vascular circulation of COVID-19 disease and the occurrence of a retinal vascular disease, as presented in this case.

Retinal findings possibly associated with COVID-19 infection have been reported as hyper-reflective lesions at the level of ganglion cell and inner plexiform, or signs of cotton wool spots and microhaemorrhages along the retinal arcade.^10^

Further case reports have shown COVID-19 patients presented with a paracentral acute middle maculopathy and an acute macular neuroretinopathy,^11^ and a young male patient diagnosed with a Papillophlebitis.^12^

The affectation of the retinal circulation in patients with Covid-19 has been previously suggested and it reinforces the idea that the affectation is not a coincidence but rather a consequence of the disease.

The presentation of unilateral CRVO in our young and healthy patient, without any identifiable hypercoagulability risk factors, shortly after recovery from COVID-19, may imply that CRVO in this patient is due to the thromboembolic state associated with SARS-CoV-2 infection.

As the literature on human ocular manifestations of COVID-19 is still sparse, our case emphasizes the need for further investigation of ocular complication associated with this novel disease.

Finally, we emphasize the need for CRVO work-up not just during pandemic but propose that physicians should be vigilant for acute visual symptoms and looking for signs of thrombotic complications, including the ophthalmic manifestations of retinal vein and artery occlusion as a manifestation of COVID-19 patients.

## 4 Conclusion

In this case study, we present the first possible association between a suspected COVID-19 patient and unilateral CRVO in a healthy young male without any prior history or risk factors for thromboembolism. Further studies should be performed to better understand the phenomenon.

## Funding

No funding or grant support.

## Conflicts of interest

The following authors have no financial disclosures: TY, JP,RA, YP.

## Authorship

All authors attest that they meet the current ICMJE criteria for Authorship.

## Patient consent

Informed consent was obtained in writing from the patient for the use of his health information.
